# A study on speciation and antifungal susceptibility pattern of Candida isolates from HIV patients with oropharyngeal candidiasis and correlation with CD4 count

**DOI:** 10.1186/1471-2334-12-S1-P19

**Published:** 2012-05-04

**Authors:** V Kalpanadevi, S Geethalakshmi, G Sumathi

**Affiliations:** 1 Department of Microbiology, Madras Medical College, Chennai, India

## Background

Oral candidiasis is a clinical predictor for progression to AIDS. Antifungal drug resistance is becoming a major problem with this immunedepleted population. Considering the above facts, the study was conducted to speciate and to determine the susceptibility pattern of the *Candida* isolates from HIV patients with oral candidiasis and to correlate it with the CD4 count of the patients.

## Materials and methods

Samples collected from (n=150) the lesion using sterile cotton swabs in HIV patients with oral candidiasis. Isolation and speciation were done by standard mycological procedures. Antifungal susceptibility was determined by Microbroth dilution method, as per the CLSI guidelines. Estimation of CD4+ T lymphocyte of the patients was done by FACS count system.

## Results

Of the 150 samples, two revealed a mixture accounting for the 152 isolates. *Candida albicans* 118 (78%) was the most common species followed by *Candida tropicalis* 17 (11%), *Candida krusei* 8 (5%), *Candida parapsilosis* 6 (4%), *Candida glabrata* 2 (1%) and *Candida guilliermondi* 1 (1%) . By microbroth dilution 18 (11.8%) isolates were fluconazole resistant, 23 (15.1%) were itraconazole resistant and all were amphotericin susceptible. Of the 150 patients, 106 (70.6%) had CD4 count <200 cells/ µl. Azole resistant was more common in patients with CD4 count <200 cells/ µl.

## Conclusion

*Candida albicans* is the most frequently isolated species. Non- *albicans Candida species* are emerging as important pathogens with increasing rates of azole resistance and with increased immunosuppression. This emphasizes the need for speciation and determination of susceptibility pattern of the *Candida* isolates from HIV patients with oropharyngeal candidiasis.

